# Obturator Nerve Block Is Associated with Improved Histopathological Specimen Quality and Fewer Perioperative Complications During TURBT for Lateral Bladder Wall Tumors

**DOI:** 10.3390/jcm15145473

**Published:** 2026-07-13

**Authors:** Dragoș Florin Vasile, Nelu Vivi Călina, Mihnea Meșină, Mihai Alexandru Radu, George G. Mitroi, Alex Emilian Stepan, Cosmin Vasile Obleagă, Dragoș George Popa, Stan Marius Doru, George F. Mitroi

**Affiliations:** 1 Department of Urology, University of Medicine and Pharmacy of Craiova, 200349 Craiova, Romania; dragos.vasile@umfcv.ro (D.F.V.); mihneamesina@yahoo.com (M.M.); 28radu.mihai@gmail.com (M.A.R.); george.mitroi@umfcv.ro (G.F.M.); 2 Department of Anesthesiology and Intensive Care, Clinical County Emergency Hospital of Craiova, 200349 Craiova, Romania; calinavivi@yahoo.com; 3 Department of Dermatology, University of Medicine and Pharmacy of Craiova, 200349 Craiova, Romania; george.mitroi94@umfcv.ro; 4 Department of Pathology, University of Medicine and Pharmacy of Craiova, 200349 Craiova, Romania; astepan76@yahoo.com; 5 Department of Surgical Oncology, University of Medicine and Pharmacy of Craiova, 200349 Craiova, Romania; 6 Department of Plastic Surgery, University of Medicine and Pharmacy of Craiova, 200349 Craiova, Romania; dragos.popa@umfcv.ro; 7 Department of Urology, Clinical County Emergency Hospital of Constanța, 900591 Constanța, Romania; stan.marius1988@yahoo.com

**Keywords:** bladder cancer, transurethral resection of bladder tumor, obturator nerve block, non-muscle-invasive bladder cancer, specimen quality, detrusor muscle

## Abstract

**Background/Objectives**: Transurethral resection of bladder tumors (TURBT) is the standard for diagnosing and treating non-muscle-invasive bladder cancer. For lateral bladder wall tumors, obturator nerve stimulation can trigger sudden adductor contractions, raising the risk of perforation, hemorrhage, incomplete resection, and poor specimen quality. We evaluated the impact of obturator nerve block (ONB) on specimen quality and perioperative complications. **Methods**: In this single-center retrospective study, patients with lateral wall tumors treated by TURBT between October 2022 and December 2024 were divided into an ONB group (spinal anesthesia plus ONB) and a non-ONB group (spinal anesthesia alone). Specimen quality, perioperative complications, and 12-month recurrence were analyzed. **Results**: In this retrospective cohort of 219 patients (135 ONB, 84 non-ONB), high-quality specimens were more frequent with ONB (71.1% vs. 35.7%, *p* < 0.001). No perforations occurred with ONB versus 5 (6.0%) without (*p* = 0.008); hematuria (11.1% vs. 28.6%, *p* = 0.002) and 12-month recurrence (4.4% vs. 16.7%, *p* = 0.005) were also lower. **Conclusions**: ONB added to spinal anesthesia during TURBT for lateral wall tumors was associated with improved specimen quality and fewer perioperative complications. The lower recurrence rate should be considered hypothesis-generating, given the retrospective design and the small number of recurrence events; prospective studies are needed.

## 1. Introduction

Bladder cancer is the seventh most common cancer diagnosed in men worldwide and the ninth when both sexes are considered [1]. In urological practice, approximately 75% of patients present with non-muscle-invasive forms, corresponding to stages Ta and T1, and this proportion is even higher in younger patients under 40 years of age. At these stages, transurethral resection of the bladder tumor (TURBT) is the standard method for both diagnosis and treatment [2,3]. The quality of the specimens obtained by endoscopic resection plays an essential role in correct disease staging and directly influences subsequent therapeutic management, including the indication for intravesical instillations, re-TURBT, or radical surgery. The presence of detrusor muscle fibers in the histopathological specimen is considered an important indicator of a complete, high-quality resection [4]. Their absence is a major factor for staging error, particularly understaging, with a potential negative impact on oncological outcomes [5,6,7,8]. In addition, coagulation artifacts may compromise the assessment of muscle invasion and resection margins. Thus, both the quality of the surgical procedure and the handling, fixation, and processing of specimens are decisive for obtaining a complete and accurate histopathological report [9].

The obturator reflex, caused by stimulation of the obturator nerve during TURBT for tumors located on the lateral bladder wall, produces sudden contractions of the thigh adductor muscles. This reaction may favor bladder perforation, intraoperative hemorrhage, and the retrieval of poor-quality tissue fragments, affecting both the safety of the procedure and the accuracy of histopathological evaluation [10,11,12]. The incidence of the obturator reflex is reported variably, particularly for tumors on the lateral wall, and has been associated with an increased risk of intraoperative complications and postoperative tumor recurrence [12,13]. Obturator nerve block combined with spinal anesthesia has been proposed as an effective method to prevent adductor contractions and reduce the risk of bladder perforation during TURBT [14,15,16]. Ultrasound- and/or nerve-stimulator-guided techniques can increase the precision of nerve localization and reduce the risk of puncture-related complications [17,18,19]. Nevertheless, data on the direct impact of obturator nerve block on the quality of the histopathological fragments obtained remain limited.

In this context, the aim of the present study was to objectively evaluate the influence of obturator nerve block on the quality of specimens obtained by TURBT and on the intraoperative safety profile, compared with the exclusive use of spinal anesthesia.

## 2. Materials and Methods

### 2.1. Study Design and Patients

We conducted a retrospective study that included all patients admitted to the Department of Urology of the Clinical County Emergency Hospital of Craiova between October 2022 and December 2024, diagnosed with bladder neoplasms identified by ultrasound and confirmed cystoscopically and histopathologically, located on the lateral bladder walls—a region where the risk of obturator reflex is considered significant. We evaluated demographic data, medical history and risk factors associated with bladder cancer, tumor size, invasion, degree of differentiation, histopathological specimen quality, and postoperative complications. Patients included in the final analysis were followed by imaging and cystoscopy for up to 12 months after resection and received oncological treatment according to current guidelines, as indicated by the multidisciplinary team. A single immediate postoperative intravesical instillation of epirubicin was administered, in accordance with current guideline recommendations for non-muscle-invasive bladder cancer, except in patients with intraoperative bladder perforation or clinically significant postoperative bleeding.

Patients aged ≥18 years, with American Society of Anesthesiologists (ASA) physical status I–III, preoperatively diagnosed with primary bladder tumors located on the lateral bladder walls, identified by imaging and confirmed cystoscopically, with an indication for TURBT under spinal anesthesia, were included. Eligible patients were those in whom the lesions were considered endoscopically resectable and preoperatively compatible with suspicion of non-muscle-invasive bladder cancer.

Patients who presented local infection at the puncture site, known allergy to local anesthetics, a history of major pelvic surgery with altered regional anatomy, pre-existing obturator neuropathy or motor deficit, inability to maintain adequate intraoperative positioning, or severe obesity impairing identification of anatomical landmarks were excluded.

The Ethics Committee of the Clinical County Emergency Hospital of Craiova approved this study (protocol code 27689; approved 29 May 2026) on the following grounds: (1) data were collected in a retrospective, observational, descriptive, and non-experimental study; (2) the study did not interfere with current medical care; (3) no substances were administered to patients and no biological samples were collected within the study; and (4) data were collected and analyzed anonymously, so that the confidentiality of patient data was not breached.

The following variables were collected: (1) baseline characteristics (age, sex, and comorbidities); (2) characteristics of the bladder neoplasm (location, tumor stage, type of surgical procedure, and intraoperative (bladder perforation) and postoperative (hematuria and urinary tract infection) complications); (3) histopathological specimen quality (Table 1); and (4) ultrasound and cystoscopic re-evaluation at a minimum of 12 months. Tumor stage was assessed using the 8th edition of the TNM Classification of Malignant Tumours of the Union for International Cancer Control [7]. Patients were divided into two groups according to obturator reflex control; this was performed depending on perioperative logistic availability and on the experience of the anesthesiologist, and resections were performed by a team of urologists experienced in these procedures. Because group allocation was not randomized but determined by these logistic and operator-related factors, the possibility of selection bias cannot be excluded; this is addressed further in the Limitations section.

### 2.2. Histopathological Quality Assessment

From a histopathological perspective, the quality of the fragments obtained by TURBT is essential for correct disease staging. An adequate fragment must allow complete evaluation of the tumor architecture and include the detrusor muscle, a key element for assessing the depth of invasion. The presence of detrusor is considered a surrogate marker of resection quality and allows reliable differentiation between non-muscle-invasive and muscle-invasive forms [4]. Thermal artifacts induced by electrocautery may compromise histological interpretation by altering tissue morphology and masking anatomical planes, limiting the evaluation of invasion and resection margins. Fragments lacking detrusor or with extensive artifacts are considered diagnostically suboptimal and are associated with an increased risk of understaging and early tumor recurrence. Therefore, the assessment of fragment quality must be systematically based on the presence of detrusor, architectural integrity, and the degree of coagulation artifacts (Table 1) [4,5]. This evaluation was performed by two pathologists experienced in urological pathology, as is standard practice in the urology department where the study was conducted; both pathologists were blinded to the anesthesia technique and to the patients’ study-group allocation, and the final quality category was assigned by consensus.

### 2.3. Anesthesia and Surgical Technique

In the non-ONB group, TURBT was performed under standard spinal anesthesia [20], in the lithotomy position, using a bipolar resectoscope (Karl Storz SE & Co. KG, Tuttlingen, Germany; 26 Fr, AUTOCON III 400, standard bipolar loop) with a continuous isotonic saline irrigation system. The procedure was carried out under standardized conditions, observing the intraoperative measures recommended to reduce the risk of obturator reflex: limiting diathermy current intensity, avoiding bladder overdistension, and consistently using the bipolar technique. These measures, frequently reported in the literature on the prevention of the “obturator jerk” during TURBT, have proven effective in reducing the incidence of adductor contractions and bladder perforations [14,16].

**Figure 1:**
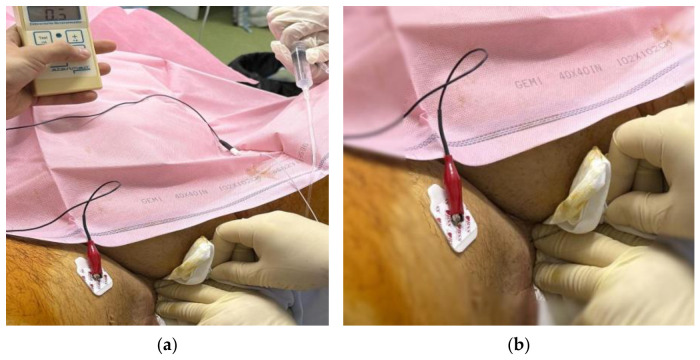
(**a**,**b**) Technique for performing the obturator nerve block (ONB).

In the ONB group, spinal anesthesia was combined with an obturator nerve block (ONB) performed prior to resection [21]. With the patient in the lithotomy position, the obturator nerve was localized using anatomical landmarks and a nerve-stimulator-guided technique, with the aim of blocking the obturator nerve and abolishing the adductor reflex (Figure 1a,b).

For confirmation of the motor response and optimization of needle positioning, neurostimulation was used: initial stimulation at 2–3 mA (2 Hz, 0.1–0.3 ms), followed by gradual current reduction to 0.3–0.5 mA before injection, with disappearance of adductor contractions as a sign of block efficacy. These settings are those recommended for the classic neurostimulator technique in ONB. The equipment used was a Stimuplex HNS 12 neurostimulator (B. Braun Melsungen AG, Melsungen, Germany), with current adjustable between 0 and 5 mA and selectable pulse duration (0.1/0.3/1.0 ms), together with 22G insulated needles (Stimuplex A/D, B. Braun Melsungen AG, Melsungen, Germany) dedicated to peripheral nerve blocks. The local anesthetic was chosen according to the estimated procedure duration: for procedures lasting <2 h, mepivacaine 1–2% or lidocaine 1–2% (5–7 mL per nerve branch) was used, whereas for blocks of extended duration, bupivacaine 0.25–0.5% or ropivacaine 0.25–0.5% was used, in accordance with the literature [22]. TURBT was subsequently performed under the same technical conditions as in the non-ONB group.

### 2.4. Statistical Analysis

A comprehensive database incorporating all variables of interest was built using Microsoft Excel 2019 MSO (version 2304, Build 16.0.16327.20200; Microsoft Corp., Redmond, WA, USA). Detailed statistical analyses were performed using MedCalc software (version 20.218; MedCalc Software Ltd, Ostend, Belgium). Frequencies were expressed as absolute numbers and percentages. Continuous variables, which were not normally distributed, were compared using the Mann–Whitney U test, and categorical variables using the chi-square test with Yates’ continuity correction for 2 × 2 tables; the Fisher exact test was used instead of the chi-square test when an expected cell frequency was below 5. A *p*-value < 0.05 was considered statistically significant. To determine whether the associations between obturator nerve block and the study outcomes were independent of potential confounders, multivariable binary logistic regression models were constructed for the primary endpoint (high-quality, Quality 1, specimen) and for postoperative hematuria and 12-month recurrence. The specimen-quality model was adjusted for age, tumor size (>3 cm vs. ≤3 cm), tumor stage (T1 vs. Ta), and tumor grade (G2 and G3 vs. G1); because all female patients and all active smokers in the ONB group had high-quality specimens (complete separation), sex and smoking status could not be entered into this model, and a Firth penalized-likelihood logistic regression was used as a sensitivity analysis. In view of the limited number of events, the hematuria model was adjusted for tumor size and stage, and the 12-month recurrence model was adjusted for tumor stage and is reported as exploratory. As all resections were performed by the same surgical team, operator was not included as a covariate. Adjusted odds ratios (aOR) with 95% confidence intervals (CI) are reported.

## 3. Results

**Figure 2:**
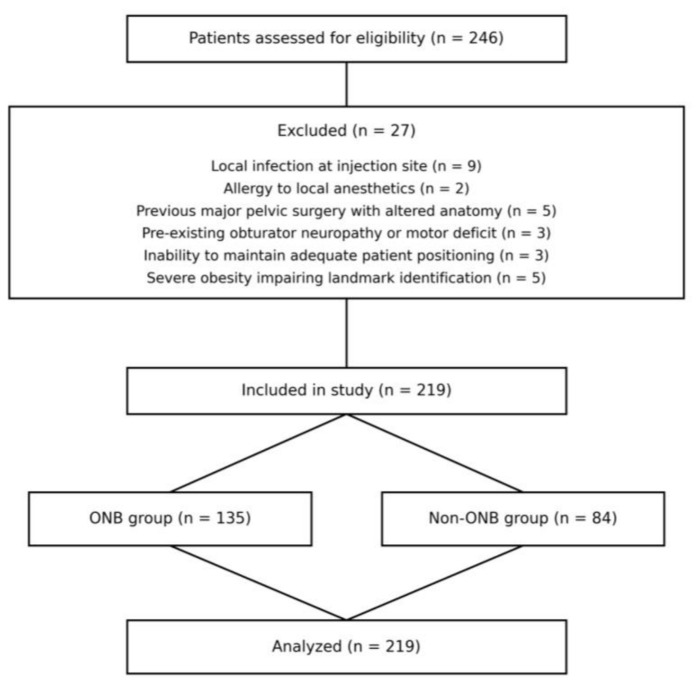
Flow diagram of the patient-selection process.

After applying the exclusion criteria, 27 of the 246 patients were excluded, and 219 patients remained eligible for the final analysis (Figure 2). Patients were assigned to two study groups: ONB (n = 135), in which obturator nerve block was performed in combination with spinal anesthesia, and non-ONB (n = 84), in which only standard spinal anesthesia was performed, without obturator block (Table 2).

Baseline demographic and clinicopathological characteristics were comparable between the two groups, with no statistically significant differences in age, body mass index, sex distribution, smoking status, alcohol consumption, tumor size, or tumor grade.

The median age was 73 years in the ONB group and 72 years in the non-ONB group (*p* = 0.220). Tumors larger than 3 cm were identified in 9.6% of patients in the ONB group and 13.1% in the non-ONB group (*p* = 0.318). Although tumor size did not differ significantly between groups, large bladder tumors are generally associated with technically more difficult resections, an increased risk of tissue fragmentation, and the possibility of incomplete resection—factors that may contribute to early recurrence. In this context, effective obturator reflex control may play an important role in optimizing resection quality, particularly for tumors located on the lateral bladder wall.

Statistically significant differences were observed between the two groups regarding the histopathological quality of resection specimens (*p* < 0.001). High-quality specimens (Quality 1) were obtained significantly more often in the ONB group than in the non-ONB group (71.1% vs. 35.7%). Conversely, intermediate-quality specimens (Quality 2) were more frequent in the non-ONB group (41.7% vs. 20.7%), and poor-quality fragments (Quality 3) were identified in 22.6% of non-ONB patients compared with only 8.1% in the ONB group.

The use of obturator nerve block was associated with a significant reduction in the rate of intraoperative bladder perforation. No perforations were recorded in the ONB group, compared with 5 cases (6.0%) in the non-ONB group (*p* = 0.008). All five perforations were managed conservatively by urinary catheter drainage, without the need for open or laparoscopic repair. Postoperative hematuria was also significantly less frequent in the ONB group than in the non-ONB group (11.1% vs. 28.6%, *p* = 0.002). Although urinary tract infections were less frequent in the ONB group (4.4% vs. 11.9%), this difference did not reach statistical significance (*p* = 0.073).

At the 12-month re-evaluation, the tumor recurrence rate was significantly lower in the ONB group than in the non-ONB group (4.4% vs. 16.7%, *p* = 0.005). Given the limited number of recurrence events, this particular finding was further examined only in an exploratory adjusted model (Table 3) and should be interpreted with caution (see Discussion and Limitations).

In multivariable analysis, obturator nerve block remained independently associated with a higher likelihood of obtaining a high-quality (Quality 1) specimen (aOR 4.80, 95% CI 2.63–8.75, *p* < 0.001) after adjustment for age, tumor size, stage, and grade; among the covariates, only high tumor grade (G3) was independently associated with specimen quality (aOR 2.87, 95% CI 1.05–7.81, *p* = 0.040) (Table 3). A Firth penalized-likelihood sensitivity analysis yielded a consistent estimate for ONB (aOR 4.54, 95% CI 2.50–8.26). Obturator nerve block also remained independently associated with lower odds of postoperative hematuria (aOR 0.31, 95% CI 0.15–0.64, *p* = 0.001) after adjustment for tumor size and stage. In an exploratory model adjusted for tumor stage, ONB was associated with lower 12-month recurrence (aOR 0.23, 95% CI 0.09–0.63, *p* = 0.004); given the small number of recurrence events (n = 20), this estimate should be regarded as hypothesis-generating.

## 4. Discussion

Demographic and clinicopathological characteristics were comparable between the two groups, with no statistically significant differences in age, sex, tumor size, tumor grade, or tumor stage. This supports the comparability of the cohorts with respect to the measured baseline variables; however, because the study is retrospective and non-randomized, residual confounding by unmeasured factors cannot be excluded.

Tumor size is an important factor in the technical complexity of TURBT, with large tumors frequently associated with an increased risk of tissue fragmentation, coagulation artifacts, and incomplete resection. In our study, the distribution of tumor size was comparable between the two groups, suggesting that the observed differences in specimen quality and perioperative complications were more likely influenced by obturator reflex control than by tumor size.

Particularly for tumors located on the lateral bladder wall, increased size may accentuate the technical difficulty of resection by requiring more extensive maneuvers and prolonged exposure to electrocautery, increasing susceptibility to adductor contractions and tissue trauma. Consistent with previously reported data, tumor size may influence the technical difficulty of TURBT and resection quality, particularly by increasing the risk of tissue fragmentation and extensive coagulation artifacts [4,6].

The obturator reflex causes sudden, uncontrolled contractions of the adductor muscles during resection, affecting the stability of the operative field and the precision of endoscopic maneuvers. These contractions may favor tissue fragmentation, charring, and the appearance of extensive thermal artifacts, limiting preservation of the histopathological specimen architecture [10,14]. In our study, the ONB group showed a significantly higher proportion of high-quality specimens and an important reduction in fragments considered suboptimal for histopathological evaluation. Consistent with previous studies, the obturator reflex represents an important factor of intraoperative instability during TURBT for lateral bladder wall tumors, influencing both the safety of the procedure and resection quality [23,24].

The quality of histopathological specimens is particularly important in the evaluation of tumors with suspected muscle invasion, because the presence and integrity of the detrusor muscle are essential for correct staging. Fragments affected by extensive thermal artifacts or tissue fragmentation may limit the assessment of invasion depth and favor understaging of the disease. In this context, the higher frequency of high-quality specimens observed in the ONB group may have important implications for the accuracy of histopathological evaluation and subsequent therapeutic management. This aspect is relevant for avoiding understaging, since reliable identification of the detrusor muscle allows correct exclusion of muscle invasion and the establishment of appropriate therapeutic management [4,5,25].

Although the incidence of urinary tract infections was lower in the ONB group, the difference did not reach statistical significance. Nevertheless, the lower rate of bladder perforation and postoperative hematuria observed in patients who received obturator nerve block may suggest a lower degree of tissue trauma and mucosal injury, with a possible favorable impact on the postoperative inflammatory and infectious profile [10,14]. Although the tumor recurrence rate was lower in the ONB group, these results should be interpreted with caution, given the relatively short follow-up period and the small number of recurrence events and the absence of adjustment for second (re-)transurethral resection, a strong determinant of recurrence in non-muscle-invasive bladder cancer. Effective obturator reflex control may contribute not only to reducing intraoperative complications but also to optimizing the oncological quality of TURBT by yielding better-preserved histopathological fragments more suitable for staging.

### Limitations

The retrospective, single-center design may introduce selection bias and limits the generalizability of the results. The follow-up period was relatively short, limited to 12 months, and multivariable logistic regression confirmed the independence of the main associations; however, sex and smoking status could not be entered into the specimen-quality model because of complete separation (all female patients and all active smokers in the ONB group had high-quality specimens), and the 12-month recurrence model was limited by the small number of events and remained exploratory. In addition, allocation to the two groups was not randomized but determined by perioperative logistic availability and anesthesiologist experience. Apart from the five non-ONB patients with intraoperative bladder perforation, in whom the immediate instillation was withheld in accordance with guideline recommendations, all patients received the same single immediate postoperative epirubicin instillation. Although this small imbalance could in principle have contributed to the higher recurrence observed in the non-ONB group, it is unlikely to fully account for the difference. Data on second (re-)TURBT were also not available for adjustment; given the small number of recurrence events and the short follow-up, the lower 12-month recurrence observed in the ONB group should be regarded as hypothesis-generating rather than confirmatory. Nevertheless, baseline demographic and clinicopathological characteristics were comparable between the two groups, reducing the likelihood of major confounding factors. In addition, the assessment of histopathological specimen quality may carry a degree of interobserver subjectivity. Because a single consensus quality score was recorded per specimen, formal interobserver agreement (Cohen’s kappa) was not quantified. Despite these limitations, the results suggest a consistent association between obturator reflex control and improved TURBT quality.

## 5. Conclusions

Obturator nerve block combined with spinal anesthesia during TURBT for lateral bladder wall tumors was associated with significantly improved histopathological specimen quality and reduced intraoperative complications. Patients in the ONB group showed a higher frequency of high-quality fragments, with preserved tissue architecture and the presence of detrusor muscle—aspects essential for correct staging and adequate oncological evaluation.

Effective obturator reflex control may contribute not only to increased procedural safety but also to optimizing the oncological quality of TURBT by facilitating a more stable and precise resection. By reducing adductor contractions and increasing intraoperative stability, obturator nerve block may enable a more precise resection, with a favorable impact on histopathological evaluation and potentially on the quality of oncological treatment.

## Figures and Tables

**Table 1:** Histopathological criteria for assessing the quality of bladder tumor resection fragments.

Quality Category	Histopathological Criteria	Diagnostic Value
Quality 1 (good)	Well-preserved tissue architecture; minimal or absent thermal artifacts; clear identification of the urothelial tumor, lamina propria, and presence of the detrusor muscle layer	Optimal for correct staging and grading; reliable assessment of invasion depth
Quality 2 (intermediate)	Moderate coagulation artifacts, with partial preservation of tissue architecture; detrusor muscle layer present but fragmented or partially masked	Acceptable for diagnosis and grading; limitations in precise assessment of invasion
Quality 3 (poor)	Extensive thermal artifacts, tissue fragmentation, or charring; absence of the detrusor muscle layer	Reduced diagnostic value; increased risk of pathological understaging and need for re-resection

The primary endpoint of the study was the proportion of high-quality histopathological specimens, defined as Quality 1 fragments with preserved tissue architecture, minimal or absent thermal artifacts, and identifiable detrusor muscle. Secondary endpoints included intraoperative bladder perforation, postoperative hematuria, urinary tract infection, and 12-month tumor recurrence.

**Table 2:** Clinicopathological characteristics of patients in the ONB and non-ONB groups.

Parameter	Total (%)	ONB	Non-ONB	*p*-Value
No. of cases	219 (100.0%)	135 (61.6%)	84 (38.4%)	
Age, years	73 (66–75)	73 (68–75)	72 (65.75–75)	0.220 ^b^
BMI, kg/m^2^	26.57 (24.22–29.41)	26.64 (24.22–29.41)	26.39 (24.22–29.49)	0.999 ^b^
Sex (%)				0.330 ^a^
Male	173 (79.0%)	110 (81.5%)	63 (75.0%)	
Female	46 (21.0%)	25 (18.5%)	21 (25.0%)	
Smoking status	72 (32.9%)	47 (34.8%)	25 (29.8%)	0.531 ^a^
Alcohol consumption	137 (62.6%)	88 (65.2%)	49 (58.3%)	0.381 ^a^
Tumor size (%)				0.318 ^a^
≤3 cm	195 (89.0%)	122 (90.4%)	73 (86.9%)	
>3 cm	24 (11.0%)	13 (9.6%)	11 (13.1%)	
Tumor grade (%)				0.892 ^a^
G1	52 (23.7%)	32 (23.7%)	20 (23.8%)	
G2	69 (31.5%)	43 (31.9%)	26 (31.0%)	
G3	98 (44.8%)	60 (44.4%)	38 (45.2%)	
Tumor stage (%)				0.981 ^a^
Ta	78 (35.6%)	48 (35.6%)	30 (35.7%)	
T1	141 (64.4%)	87 (64.4%)	54 (64.3%)	
Histopathological quality of resection specimen (%)				<0.001 ^a,^*
Quality 1	126 (57.5%)	96 (71.1%)	30 (35.7%)	
Quality 2	63 (28.8%)	28 (20.7%)	35 (41.7%)	
Quality 3	30 (13.7%)	11 (8.1%)	19 (22.6%)	
Complications (%)				
Perforation	5 (2.3%)	0 (0.0%)	5 (6.0%)	0.008 ^c,^*
Hematuria	39 (17.8%)	15 (11.1%)	24 (28.6%)	0.002 ^a,^*
Urinary tract infection	16 (7.3%)	6 (4.4%)	10 (11.9%)	0.073 ^a^
Follow-up (%)				
12-month recurrence	20 (9.1%)	6 (4.4%)	14 (16.7%)	0.005 ^a,^*

* Statistically significant (*p* < 0.05); ^a^ chi-square test; ^b^ Mann–Whitney U test; ^c^ Fisher exact test; ONB, obturator nerve block; BMI, body mass index.

**Table 3:** Crude and adjusted associations between obturator nerve block and study outcomes (multivariable binary logistic regression).

Outcome (ONB vs. Non-ONB)	Crude OR (95% CI)	*p*	Adjusted OR (95% CI)	*p*
High-quality (Quality 1) specimen	4.43 (2.48–7.92)	<0.001	4.80 (2.63–8.75)	<0.001
Postoperative hematuria	0.31 (0.15–0.64)	0.001	0.31 (0.15–0.64)	0.001
12-month recurrence (exploratory)	0.23 (0.09–0.63)	0.004	0.23 (0.09–0.63)	0.004

OR, odds ratio; CI, confidence interval; ONB, obturator nerve block. The specimen-quality model was adjusted for age, tumor size, stage, and grade; the hematuria model for tumor size and stage; the exploratory recurrence model for tumor stage. Sex and smoking were not estimable in the specimen-quality model owing to complete separation.

## Data Availability

The data presented in this study are available on request from the corresponding author.
